# Hysteroscopic diode laser myolysis: from a case series to literature review of incisionless myolysis techniques for managing heavy menstrual bleeding in premenopausal women

**DOI:** 10.1007/s00404-023-07218-y

**Published:** 2023-10-13

**Authors:** Salvatore Giovanni Vitale, Stefania Saponara, Gilda Sicilia, Marko Klarić, Felice Sorrentino, Maurizio Nicola D’Alterio, Luigi Nappi, Stefano Angioni

**Affiliations:** 1 Division of Gynecology and Obstetrics, Department of Surgical Sciences, University of Cagliari, Cagliari, Italy; 2 Clinical Hospital Center of Rijeka, Department of Obstetrics and Gynaecology, Rijeka, Croatia; 3 Department of Medical and Surgical Sciences, Institute of Obstetrics and Gynaecology, University of Foggia, Foggia, Italy

**Keywords:** Hysteroscopic laser myolysis, Heavy menstrual bleeding, Hysteroscopy

## Abstract

**Purpose:**

This case series examined the safety and effectiveness of hysteroscopic myolysis using laser-induced interstitial thermo-therapy (LITT) for treating heavy menstrual bleeding (HMB) in premenopausal women with FIGO type 1 or 2 uterine fibroids, not planning for future fertility. Additionally, a comprehensive review of innovative, minimally invasive, incisionless myolysis techniques was conducted.

**Methods:**

Women with HMB, sonographically diagnosed with a single FIGO type 1 or 2 fibroid, underwent hysteroscopic myolysis using the Leonardo^®^ diode laser. Effectiveness was assessed via transvaginal ultrasound measurement of myoma size, volume and vascularization pre and post-procedure. Moreover, we also evaluated any improvements in symptoms using the Pictorial Blood Loss Assessment Chart (PBAC score) scores.

**Results:**

The procedure resulted in significant HMB reductions and noticeable fibroid size, volume, and vascularization decrease in all three patients, with no reported complications. The literature review revealed both advantages and limitations of the minimally invasive, incisionless myolysis techniques.

**Conclusions:**

Hysteroscopic laser myolysis is a safe and effective therapeutic intervention for patients experiencing HMB, diagnosed with FIGO type 1 or 2 fibroids, and not planning for future fertility. The procedure resulted in significant reductions in menstrual blood loss and fibroid size. Despite the promising results, it is essential to note the limitations of this report, including its case series design, a small number of patients, and a short follow-up period. Further research is necessary to confirm these results.

## Introduction

Uterine leiomyomas, or uterine fibroids, are common benign smooth muscle tumors that typically manifest during a woman’s reproductive years [1]. These tumors significantly influence women’s health worldwide, with predisposing factors such as African ethnicity, nulliparity, and obesity increasing their likelihood of occurrence [2, 3]. The estimated prevalence and incidence of uterine fibroids are high, with over 60% of African American women affected by age 35, increasing to over 80% after menopause. Similarly, about 40% of white women have fibroids by age 35, rising to 70% by age 50 [4].

Despite their high prevalence, fibroids often present asymptomatically. Only about 25% of women diagnosed with fibroids experience symptoms, but when present, they significantly impact the quality of life [2, 5, 6]. The symptoms depend on the fibroids' size, number, and location. They may manifest as heavy menstrual bleeding (HMB), anemia, dysmenorrhea, pelvic pain or discomfort, fatigue, impaired urination, and constipation [2, 3]. Among these symptoms, HMB is often the first or the only noticeable indication of fibroids in women [7, 8]. The Nice guidelines define HMB as excessive menstrual blood loss, which can profoundly affect various aspects of a woman's life [8]. More specifically, the presence of HMB can harm a woman's physical well-being, emotional state, social interactions, and material quality of life [8]. Therefore, managing HMB and other related symptoms is crucial to improving the life quality of women diagnosed with uterine fibroids.

Various treatments for fibroids and their symptomatology are available, ranging from medical and expectant management to conventional surgical options and newer, less invasive [2, 3, 9–13]. Selecting an appropriate treatment option considers various aspects, such as the size, number, and location of the fibroids, the symptom’s presence, the patient’s age, the desire for fertility preservation, and the individual patient's preference [2, 9–11]. Over the past decades, interest has increased in less invasive and fertility-preserving techniques for treating fibroids [11, 14, 15]. One such promising method is myolysis, a procedure that employs different types of energy to coagulate fibroid tissues instead of removing them entirely. This process results in the shrinkage of fibroids and consequential relief of symptoms [16]. Myolysis was introduced as an innovative procedure in the early 1990 s [16, 17]. This procedure utilized a Nd: YAG laser to penetrate the fibroid's pseudo-capsule, instigate tissue coagulation, obliterate the fibroid's blood supply, and consequently induce fibroid shrinkage, all performed via laparoscopy [15, 16].

A new development in laser technology, the diode laser, has emerged recently [18, 19]. This laser generates two wavelengths (980 + 1470 nm) from a diode semiconductor. These dual wavelengths facilitate simultaneous absorption in water (1470 nm) and hemoglobin (980 nm), ensuring high performance in cutting, vaporization, and hemostasis.

The diode laser is employed in multiple hysteroscopic surgical procedures, including metroplasty for the septate uterus, myomectomy, and polypectomies [17, 20–23]. Evidence supports its safety and efficacy, marking it as a potent tool in minimally invasive gynecologic surgery [20–26].

We present a case series of three patients diagnosed with FIGO (International Federation of Gynecology and Obstetrics) grade 1 or 2 uterine fibroids and experiencing heavy menstrual bleeding (HMB). Each patient underwent hysteroscopic myolysis, utilizing laser-induced interstitial thermotherapy (LITT) with the Leonardo^®^ Diode Laser DWLS (Biolitec^®^, Jena, Germany). We supplement our case series report with a comprehensive literature review on English-language publications regarding new minimally invasive, incisionless myolysis techniques in premenopausal women diagnosed with uterine fibroids who experienced HMB.

## Materials and methods

In this case series, we aimed to evaluate the safety and effectiveness of hysteroscopic laser myolysis in treating patients with an ultrasound diagnosed FIGO grade 1 or 2 fibroids who were experiencing heavy menstrual bleeding (HMB) and had no intention of future fertility. Complementing our case series, we conducted a comprehensive review of the literature, focusing on English-language publications exploring innovative, minimally invasive, incisionless myolysis techniques used in premenopausal women with a confirmed diagnosis of uterine fibroids experiencing heavy menstrual bleeding (HMB).

We complied with the quality standards for narrative reviews, as characterized and measured by the Scale for the Quality Assessment of Narrative Review Articles (SANRA) [27].

Our literature review involved extensive research on electronic databases such as MEDLINE and PubMed, which was supplemented by manually cross-checking the reference lists of relevant publications. The search strategy was designed with a combination of controlled vocabulary specific to each database and pertinent keyword terms. Included in the search parameters were a range of concepts, such as: ("Hysteroscopy" OR "Transvaginal" OR "Transcervical") AND ("Heavy Menstrual Bleeding" OR "metrorrhagia" OR "Hypermenorrhea") AND ("myolysis" OR "ablation" OR "coagulation") AND ("Leiomyoma" OR "leiomyomata" OR "fibroid" OR "fibroids" OR "fibromyoma" OR "myoma" OR "myomas").

We limited our literature review to observational studies and clinical trials from January 2000 until May 2023. Articles not published in English, commentaries, letters, abstracts, editorials, case reports, and case series were excluded.

### Participants

This case series report occurred at the University Hospital of Foggia, University Hospital of Cagliari and Clinical Hospital Center of Rijeka between December 2021 and August 2022. The study received approval from the Ethical Committees of all three hospitals and adhered to the standards outlined in the 1964 Helsinki Declaration and its subsequent amendments or equivalent ethical guidelines. All participants provided written informed consent before undergoing any procedures, and permission for publication was secured. The study was not advertised and no remuneration was offered to encourage patients to consent to enter or continue the investigation. The eligible participants for this report were women experiencing symptomatic myomatosis, particularly heavy menstrual bleeding (HMB), and diagnosed via sonography with a single FIGO grade 1 or 2 fibroids. These women did not have any plans for future fertility.

We excluded patients with contraindications to hysteroscopy such as cervical stenosis or active pelvic infection, and those with obesity, a history of radiation therapy, retinoblastoma, or past or current tamoxifen treatment due to their increased risk of uterine sarcoma. Other exclusion criteria included patients with fibroids FIGO class 3 or more, known gynecologic malignancy, pregnancy, desire for future fertility, and prior cervical surgery.

### Transvaginal ultrasound scanning

All patients underwent a 2D-DS (two-dimensional Doppler sonography) transvaginal ultrasound uterine evaluation in the first half of their menstrual cycle. This assessment aimed to identify the myoma's specific characteristics while excluding the presence of other possible lesions. To determine the size of the myoma, we measured three diameters: anteroposterior (*D*1), longitudinal (*D*2), and transversal (*D*3). The volume was calculated using the following equation for an ellipsoid volume: *V* = 0.5233 × *D*1 × *D*2 × *D*3.

We identified the location of the uterine fibroids using the FIGO classification by Munro et al. [28].

The Doppler ultrasound was utilized to determine the vascularization grade of the myoma based on the criteria provided by the Morphological Uterus Sonographic Assessment (MUSA) group [29]. The degree of vascularization was conveyed using a subjective color score. The amount of color within a lesion was reported using this color score (CS): 1 for no color, 2 for minimal color, 3 for moderate color, and 4 for abundant color. This score reflects a subjective evaluation of both the percentage of the lesion that is vascularized and the color hue. Expert sonographers meticulously conducted the ultrasound assessments utilizing a multifrequency transvaginal probe ranging from 5.0 to 9.0 MHz of GE Voluson E10 ultrasound machine (GE Medical Systems, Zipf, Austria).

### Hysteroscopic procedure

The myolysis procedure utilized the Leonardo^®^ Diode laser (Biolitec), which enabled laser-induced interstitial thermo-therapy (LITT). This laser fiber, specifically engineered to function via the 5 Fr working channel of Bettocchi^®^ Hysteroscope (Karl Storz, Tuttlingen, Germany) with an outer diameter of 5 mm and an inner sheath of 4.3 mm, operates using two distinct wavelengths: 1470 nm (which shows an affinity for water) and 980 nm (which targets hemoglobin). These wavelengths have different action radii; the 1470 nm wavelength vaporizes nearby tissue, while the 980 nm wavelength coagulates blood vessels over a larger area, impeding the blood supply even if myoma vaporization is incomplete.

The procedure began with the patient positioned lithotomically and a vaginoscopic no-touch technique was employed. We introduced a 30-degree hysteroscope with an outer diameter of 5 mm with an inner sheath of 4.3 mm and a 5 Fr. Working channel (Bettocchi Hysteroscope, Karl Storz, Tuttlingen, Germany) into the uterine cavity distended with saline solution. The intrauterine pressure was controlled with a pump system and maintained at 50–70 mm Hg. The procedure was performed in an outpatient setting without anesthesia. The first step was to identify and confirm hysteroscopically the location and size of the fibroid, previously diagnosed via ultrasound. Upon confirmation, the diode laser (Dwls Leonardo, BioLitec, Germany) was passed through the hysteroscope's working channel into the uterine cavity. The selected fibroid was punctured with the laser fiber until its tip reached the fibroid's center. Once assured of the laser fiber tip's correct placement, we emitted a continuous 15-W coagulative circular energy from the laser diode tip, which traveled toward the fibroid's periphery.

The procedure was deemed complete when the surgeon, based on visual inspection and estimation, determined that the majority of the fibroid had undergone coagulation. During the myolysis of the fibroid, patients were asked to report their pain levels using a 0–10 Visual Analog Scale (VAS) at three distinct time points: at the onset of the myolysis procedure, midway through, and as the myolysis was concluding. Patients were advised to communicate any feelings of warmth during the procedure, as this was an essential safety measure. Such sensations could indicate that the heat produced by the laser was nearing the myometrium surrounding the fibroid, necessitating an immediate cessation of the procedure. Post-procedure, patients were observed for 20 min before being discharged from the hysteroscopy unit.

### Follow-up

Sixty days after the procedure, participants underwent a follow-up transvaginal ultrasound examination to observe any changes in the myomas’ size, volume and vascularization. The same ultrasound specialist who had performed the initial examination conducted this follow-up ultrasound. Moreover, we also assessed any improvements in symptoms using the Pictorial Blood Loss Assessment Chart (PBAC score) [30]. Participants were asked to determine their typical menstrual bleeding using the PBAC, both before the procedure and 60 days afterward. We considered a positive change in heavy menstrual bleeding (HMB) post-myolysis procedure as a reduction of at least 50% from the initial PBAC score.

## Case series

The clinical profiles of the patients, their sonographic observations, and the outcomes of the treatment are comprehensively detailed in Tables 1, 2.

**Table 1:** Demographic and clinical characteristics of patients undergoing hysteroscopic laser myolysis

Characteristic	Case 1	Case 2	Case 3
Age (years)	43	40	45
Body mass index (kg/m^2^)	26	24	24
Ethnicity	Caucasian	Caucasian	Caucasian
Parity	3	1	1
Type of fibroid (FIGO Classification)	FIGO 2	FIGO 2	FIGO 1
Fibroid location	Posterior uterine wall	Anterior uterine wall	Anterior uterine wall

FIGO, International Federation of Gynecology and Obstetrics

**Table 2:** Comparison of procedure outcomes before and after hysteroscopic laser myolysis

Procedure outcomes	Before procedure			After procedure		
	Case 1	Case 2	Case 3	Case 1	Case 2	Case 3
Myoma size (mm)						
Anteroposterior	21	23	13	14	24	11
Longitudinal	22	31	15	13	23	9
Transversal	29	30	12	20	20	8
Myoma volume (mm^3^)	6726.4	10,852.4	1210.5	1891.56	6239.9	332.8
Color score	2	2	2	1	1	1
PBAC score	395	580	603	99	97	101

PBAC, Pictorial Blood Loss Assessment Chart International

### Case 1

**Fig. 1:**
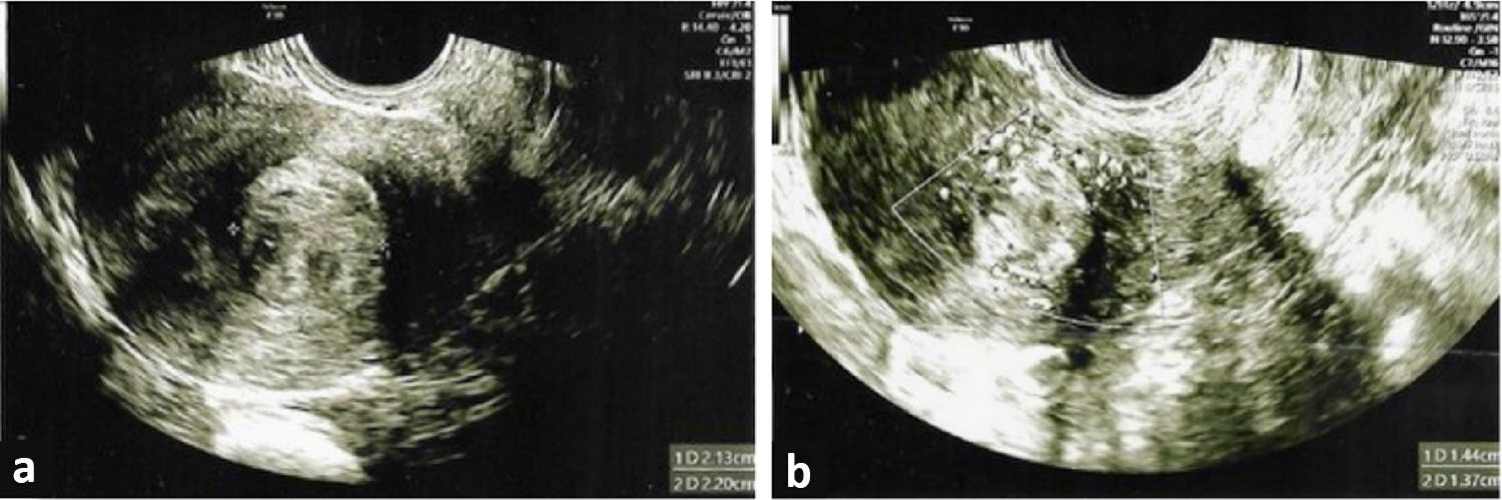
Ultrasonographic images of the fibroid in Case 1. **a** Ultrasonographic image of FIGO grade 2 fibroid on the posterior uterine wall before procedure; **b** Follow-up ultrasound image showing the reduced fibroid size post-myolysis

**Fig. 2:**
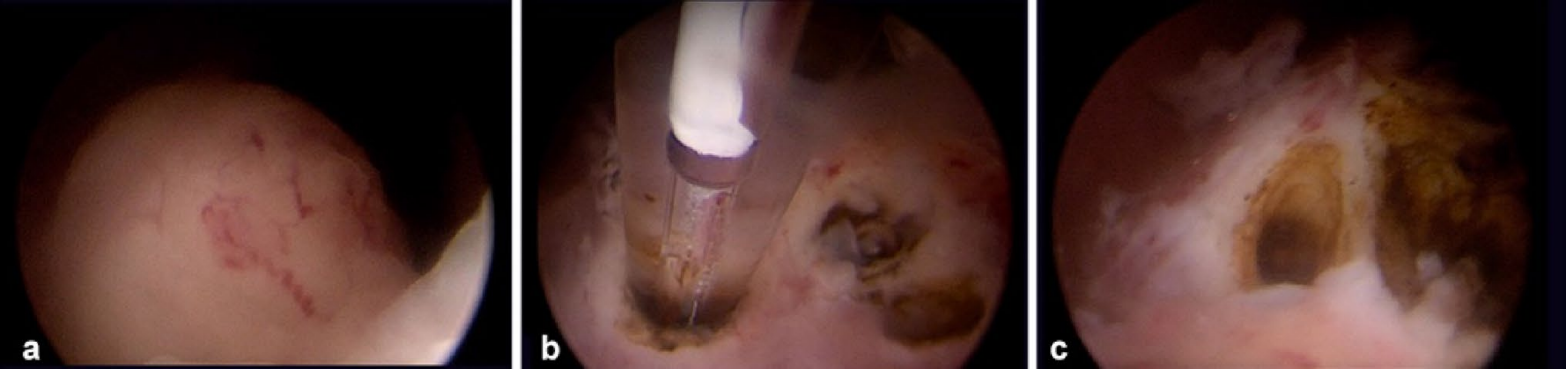
Myolysis procedure in Case 1. **a** Initial hysteroscopic view identifying the size and location of the fibroid before the procedure.; **b** insertion of the laser fiber inside the fibroid; **c** visualization of the vaporized core of the fibroid after hysteroscopic laser myolysis

A 43-year-old woman with a Body Mass Index (BMI) of 26 and a parity of three, who had not previously undergone any surgical procedures, presented with a preoperative PBAC (Pictorial Blood Loss Assessment Chart) score of 395. Other symptoms were absent. Ultrasonographic examination revealed a posterior wall fibroid measuring 21 × 22 × 29 mm, with a volume of 6726.4 mm^3^, classified as grade 2 according to the FIGO classification (Fig. 1a). The vascularization of the fibroid, assessed using Color Doppler, was categorized as CS 2 according to the Musa criteria, indicating moderate vascularization. Hysteroscopically, the position and size of the fibroid were confirmed (Fig. 2a). The laser fiber was guided to the center of the fibroid, carefully monitoring the patient's comfort throughout the process (Fig. 2b). The fibroid was effectively coagulated, as confirmed visually by the surgeon (Fig. 2c). During the myolysis procedure, which was completed without any complications, patients were asked to report their pain levels using a 0–10 Visual Analog Scale at the initiation of myolysis, midway, and upon nearing completion. The average VAS score reported during the procedure was 4. The patient was reevaluated after 60 days. The PBAC score after the procedure was 97, reflecting a reduction in menstrual blood loss by about 75%. In addition, the follow-up ultrasonographic assessment showed a decrease in the fibroid dimensions to 14 × 13 × 20 mm (Fig. 1b), corresponding to a volume of approximately 1891.56 mm^3^. This represents an approximate 72% reduction in the volume of the fibroid. The post-procedure vascularization of the fibroid was reassessed and categorized as CS 1, indicating no vascularization and showing an effective decrease in the blood supply to the fibroid.

### Case 2

**Fig. 3:**
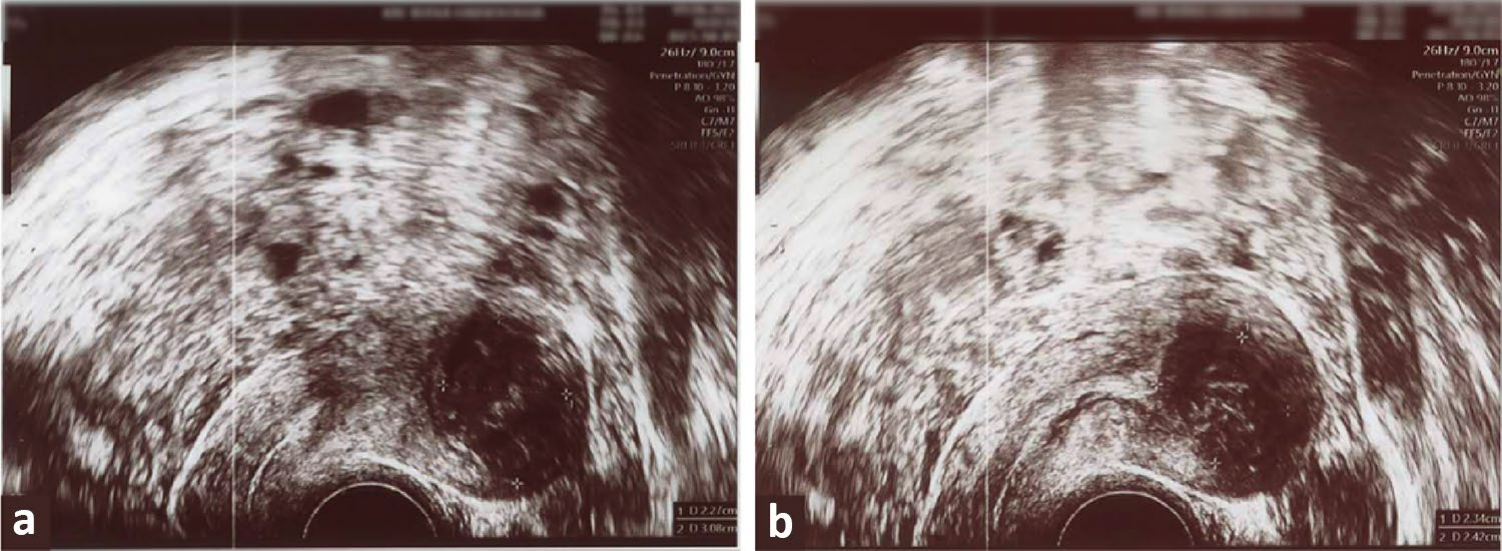
Ultrasonographic images of the fibroid in Case 2. **a** Ultrasonographic image of FIGO grade 2 fibroid on the anterior uterine wall before procedure; **b** Follow-up ultrasound image showing the reduced fibroid size post-myolysis

The second patient was a 40-year-old woman with a Body Mass Index (BMI) of 24 who had given birth once. Her Pictorial Blood Assessment Chart (PBAC) score before undergoing myolysis was 580, indicating severe HMB. No other symptoms were reported. The initial ultrasonographic evaluation identified a FIGO grade 2 fibroid on the anterior uterine wall, measuring 23 × 31 × 30 mm. (Fig. 3a) with a volume of approximately 10,852.4 mm^3^. The fibroid vascularization was categorized as CS 2 according to the Musa criteria. The hysteroscopic examination confirmed the ultrasonographic diagnosis. The surgeon could accurately position the laser fiber and induce effective coagulation of the fibroid tissue. During the myolysis procedure, executed without complications, the patient reported her pain experience via a 0–10 Visual Analog Scale. The reported average VAS score during the procedure was remarkably low at 3, indicating minimal discomfort. Upon revaluation 60 days after the procedure, the patient's PBAC score was significantly reduced to 97, indicating an approximate reduction of 83%. In addition, a follow-up ultrasound examination also revealed a decrease in fibroid size to 24 × 23 × 20 mm (Fig. 3b) with a volume of 6239.9 mm^3^, equating to a 43% volume reduction. Vascularization was reassessed as CS 1.

### Case 3

**Fig. 4:**
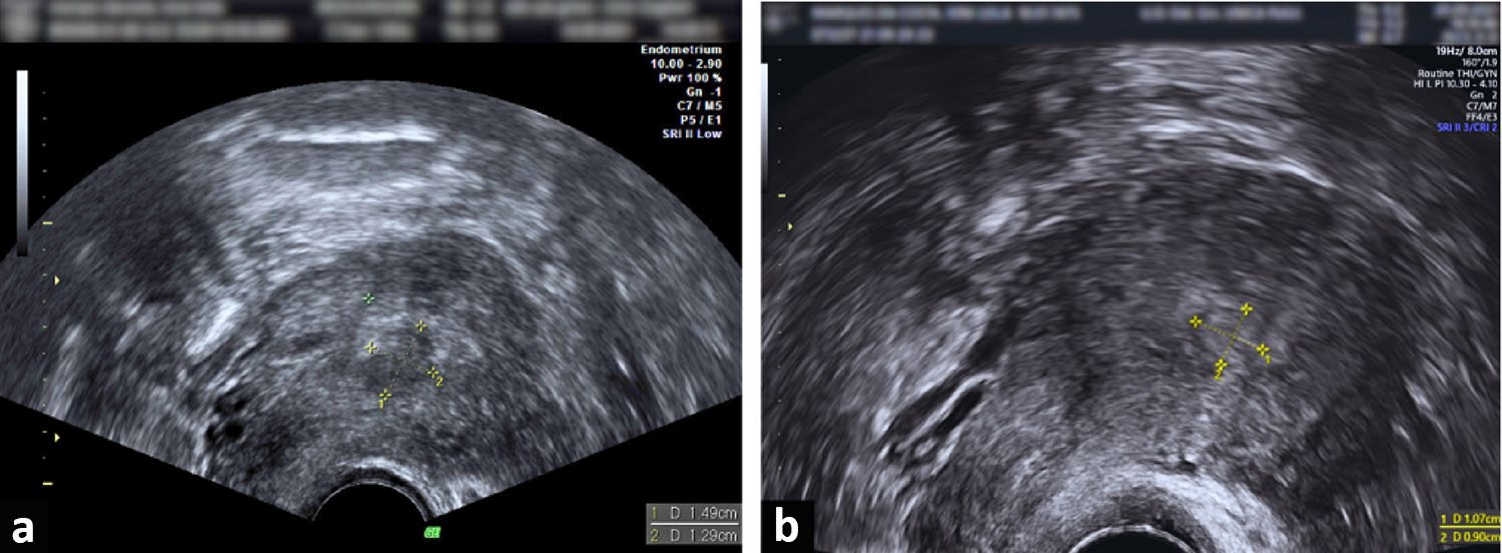
Ultrasonographic images of the fibroid in Case 3. **a** Ultrasonographic image of FIGO grade 1 fibroid in the anterior uterine wall before procedure; **b** Follow-up ultrasound image showing the reduced fibroid size post-myolysis

**Fig. 5:**
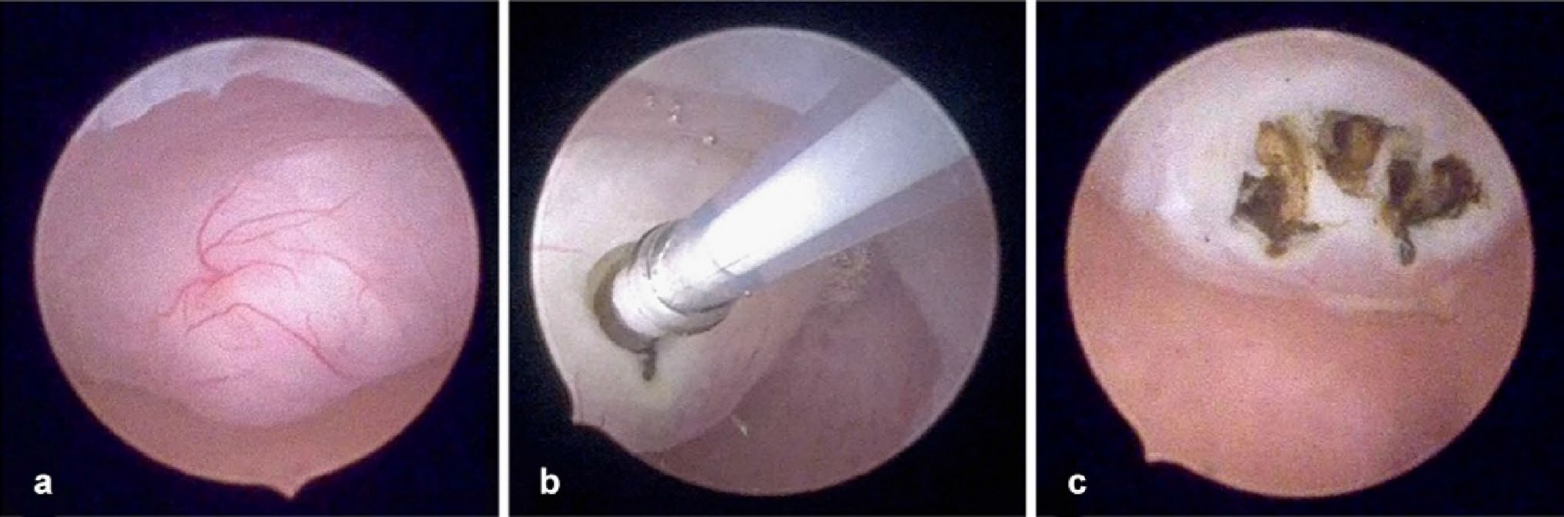
Myolysis procedure in Case 3. **a** Initial hysteroscopic view identifying the size and location of the fibroid before the procedure.; **b** insertion of the laser fiber inside the fibroid; **c** visualization of the vaporized core of the fibroid after hysteroscopic laser myolysis

The third patient was a 45-year-old woman with a BMI of 24. She had given birth once and had no chronic health conditions. An ultrasound examination revealed a fibroid in the anterior wall of her uterus, classified as FIGO grade 1 and measuring 13 × 15 × 12 mm (Fig. 4a) with a calculated volume of 1210.5 mm^3^. The fibroid showed CS 2 vascularization. Her Pictorial Blood Assessment Chart (PBAC) score before the myolysis procedure was 603, and she reported no other symptoms. Hysteroscopy confirmed the fibroid's presence, position, and size (Fig. 5a). The surgeon effectively guided the laser fiber to the core of the fibroid, ensuring adequate coagulation (Fig. 5b–c). The myolysis was carried out without any adverse events. Throughout this period, the patient was asked to periodically rate her discomfort using a 0–10 Visual Analog Scale. The average VAS score recorded during the procedure was 4, suggesting manageable discomfort. At the 60-day follow-up, her PBAC score had significantly decreased to 101, showcasing an 83% reduction in HMB. The fibroid size was measured as 11 × 9 × 8 mm on the follow-up ultrasound (Fig. 4b), with a recalculated volume of 332.8 mm^3^, indicating a volume reduction of approximately 72%. Vascularization was reassessed as CS1, indicating no vascularization.

## Discussion

Owing to their prevalence and associated symptomatology, uterine fibroids constitute a significant societal and health issue, leading to gynecological hospitalizations and around 80% of all hysterectomies [31, 32]. Evidence from a 2013 study highlighted the extent of this issue, revealing that an overwhelming 80% of women preferred less invasive alternatives for fibroid management, and 51% wished for uterine preservation. Notably, a quarter of the women surveyed delayed their treatment for up to five years [33]. Traditional therapeutic strategies like hysterectomy, myomectomy, and uterine artery embolization typically involve substantial surgical intervention and hospital stays, necessitate incisions and general anesthesia, and may lead to significant adverse events, potentially delaying a return to daily life activities [1, 2].

Hysteroscopic removal is recognized as the gold standard and most effective minimally invasive surgical approach for managing submucous fibroids [34–36]. However, it is worth noting that this procedure, particularly for G2 myomas, remains complex and should be reserved for surgeons with considerable hysteroscopy experience [35]. The potential risk of complications during hysteroscopic myomectomy is directly correlated with the intramural extension of the myoma [35, 36]. Given this context, the current therapeutic landscape for fibroid management is characterized by a pressing demand for non-invasive modalities. The ideal therapeutic procedure should preserve the uterus and provide a solution for managing intramural myomas without necessitating surgical incisions. The quest for innovative, minimally invasive fibroid management solutions among women wishing to retain their uterus has increased interest in incisionless techniques. These methods utilize various forms of energy, such as radiofrequency, focused ultrasound, microwaves, and laser technology, to heat and ablate uterine fibroids rather than physically remove them.

Radiofrequency ablation (RFA) has been established as a viable, effective treatment alternative to surgery. First reported by Lee in 2002, RFA induces coagulative necrosis in the uterine fibroid, leading to a subsequent reduction in fibroid-related symptoms [37]. Earlier versions of RFA devices required laparotomy or laparoscopy [38, 39], but advancements in sonographic guidance have enabled transcervical or transvaginal approaches, eliminating the need for surgical incisions. Several studies have validated the effectiveness of RFA in diminishing fibroid-related bleeding encompassing both transvaginal and transcervical methodologies [40–49].

Transvaginal RFA, guided by ultrasound, involves the insertion of a needle electrode into the target myoma through a needle guide attached to a transvaginal ultrasound probe. The needle electrode, linked to a generator, creates the ablation of the myoma. The procedure is performed as an outpatient protocol under mild sedation [40–44]. Kim et al. in a study involving 69 premenopausal patients, demonstrated significant improvement of menorrhagia at 1, 3, 6, and 12 months post-operation and noted no significant complications. Furthermore, three patients conceived and delivered post-procedure successfully, indicating the fertility-preserving potential of this method [40]. Similarly, Rey et al. found transvaginal radiofrequency myolysis to be a safe and effective technique for treating metrorrhagia, reducing myoma volume, and correcting anemia in 205 patients [41]. Significant findings were also reported by Yin et al. in their comprehensive 10-year retrospective cohort study involving 476 premenopausal patients, where a marked improvement in hemoglobin levels was observed 12 and 24 months post-RFA [42]. Additionally, Jiang et al. reported a significant reduction in menorrhagia scores from baseline, denoting improvement in menorrhagia at 3, 6, and 12 month follow-ups, with no major complications [43]. Lastly, a recent 2022 study demonstrated statistically significant symptomatic improvements in all analyzed bleeding variables at 0, 2, 6, and 12 months post-procedure among a cohort of 59 patients [44].

Another approach to radiofrequency ablation is the transcervical method, which utilizes systems (Sonata System or Viz Ablate System) that combine a reusable intrauterine ultrasound (IUUS) probe with a single-use disposable RFA handpiece. This approach facilitates the precise deployment of an introducer and needle electrodes into one or more targeted fibroids. Intrauterine sonography provides high-resolution imaging of the uterus and nearby structures, enabling the accuracy of the procedure [45–49]. The Fibroid Ablation Study-EU (FAST-EU) trial, a multicenter, prospective investigation conducted in the Netherlands, the UK, and Mexico, studied the impact of the Viz Ablate System on a group of women suffering from symptomatic fibroids. The results highlighted that over half (57.1%) of the patients demonstrated a significant reduction (> 50%) in their Menstrual Pictogram scores within three months [45]. This improvement trend continued, with 72.9% of women observing this reduction at the 6-month mark and 64.6% maintaining this reduction a year post-treatment [45, 46]. In another clinical trial, the SONATA trial, 86.3% of the participants experienced decreased menstrual bleeding three months post-treatment. This percentage rose to 95% at the 12 month follow-up [47]. However, a small fraction (5.5%) required surgical reintervention at the 2-year follow-up due to persistent fibroid-related bleeding [48]. As the observation period was extended to 3 years, the elective surgical reintervention rate for heavy menstrual bleeding (HMB) slightly increased to 8.2% [49]. Despite the favorable outcomes of radiofrequency ablation, certain limitations exist. These include the inability to provide a histopathological examination of the treated tissue during the procedure, the mandatory use of an ultrasound probe, and the lack of direct visualization of the fibroid and uterine cavity.

High-intensity focused ultrasound (HIFU), also known as magnetic resonance-guided focused ultrasound surgery (MRgFUS), is a non-invasive technique approved by the FDA in 2004 [50]. It employs targeted high-energy ultrasound waves to ablate fibroid tissue. These waves traverse the anterior abdominal wall, generating considerable heat at their convergence point to induce coagulative necrosis. Magnetic resonance (MR) guidance offers continuous imaging, enhancing the precision and safety of the procedure by providing live visualization of the fibroid and adjacent structures [50]. As of the current date, few studies have directly evaluated the impact of MRgFUS on fibroid-associated bleeding. [51–55]. Mascaretti et al., in a prospective cohort study, reported a significant reduction in inter-menstrual vaginal bleeding, with a majority of patients observing symptom resolution within a 10-day post-procedure window and the normalization of menstrual cycles by the 6 week mark post-treatment [51]. In a comprehensive two-year follow-up study, Lee et al. substantiated these findings. All participants reported alleviation in bleeding symptoms and 78.6% of the cohort noted substantial improvement or complete resolution [52]. Adding further weight to these findings, Leung et al. reported a notable reduction in the pictorial chart score utilized for assessing bleeding, marking a decrease from a baseline of 278.90 to 185.00 at the three-month follow-up [53]. Similarly, Xie et al. demonstrated in their study that all the participants who were anemic at baseline returned to normal hemoglobin levels at the twelve-month follow-up, providing evidence for MRgFUS’s potential impact on resolving fibroid-related anemia [54]. However, some findings hint at a possible recurrence of symptoms over time. A study by Mikami et al. indicated that, of their 29 participants, 51% reported relief from menstrual symptoms at the six-month post-MRgFUS period. Still, it was observed that symptoms worsened for 33% of these patients by the 12-month mark [55].

Percutaneous microwave ablation (PMWA) represents another viable ablative approach for fibroid management. Microwave technology, initially developed for liver tumor ablation, has been adapted to treat myomas. This technique has shown promise in treating submucosal myomas, with notable improvements observed in patients' hemoglobin levels at post-ablation intervals of 3, 6, and 12 months as evidenced by Yang et al. [56] and Liu et al. [57]. These findings underscore the potential of PMWA in resolving anemia resulting from heavy menstrual bleeding associated with fibroids. A recent innovative application of this technique has been demonstrated by Lin et al., where PMWA was monitored using a combination of transabdominal and transvaginal ultrasound (TA/TV US) [58]. Follow-up results from three months post-procedure showed considerable improvements in menstrual volume and hemoglobin levels [58]. However, the efficacy of PMWA is heavily influenced by the applicator’s angle of use, which could potentially limit the coverage and efficiency of ablation, particularly for myomas located in the sidewall [59]. Moreover, the inherent proximity of this area to the rectum might escalate the risk of injury to adjacent lesions during the procedure [59].

To address the limitations of existing techniques, the use of a diode laser working through the working canal of the Bettocchi hysteroscope has been proposed for myolysis. This approach allows for direct visualization and localization of the fibroid, and the procedure can be performed in an outpatient setting without anesthesia or mild sedation. The diode laser is currently deployed in various hysteroscopic surgical procedures, including polypectomy and metroplasty [19, 20]. This type of laser has demonstrated constant and accurate control over tissue vaporization, devoid of bleeding, regulated power of penetration or deepening, strong hemostatic capability, high safety standards, and significant patient compliance [17, 19, 20]. D'Alterio et al., in 2021, conducted an ex vivo study evaluating the effectiveness of the Dual Wavelength Diode Laser System (DWLS) in the ablation of fibroids. Myomas used in this study were procured post-total hysterectomy. The study showed promising results, with a fibroid volume reduction of 46.6% and 41.6% [25]. In this case series, we applied the same laser system, the Leonardo^®^ diode laser DWLS (Biolitec^®^, Jena, Germany), for hysteroscopic myolysis to treat patients with FIGO class 1 and 2 submucous fibroids. In all three cases, we observed successful outcomes, marked by decreased fibroid size, volume and vascularization. Additionally, the patients experienced a marked decrease in HMB, as indicated by the substantial reductions in their PBAC scores. Our results align with antecedent studies on diode laser utilization in hysteroscopic myolysis. As previously documented by Haimovich et al. [22] and Vitale et al. [60], hysteroscopic laser ablation of submucous myoma is viable, safe, and reduces fibroids-related bleeding.

By the use of the 1470 nm wavelength of the Leonardo^®^ diode laser, we successfully circumvented the postoperative complication, pain, and necrosis often associated with the use of the 800 nm laser in laparoscopic myolysis, as detailed by Donnez et al. [61]. The limitations of the 800 nm laser included suboptimal fibroid shrinkage and devascularization due to its inability to vaporize tissue; it could only coagulate it. In contrast, the laser used in our procedure specifically targets the fibroid's core, reducing its vascularization and reducing fibroid shrinkage without causing hypoxic damage or necrosis. The dual wavelengths of 1470 nm and 980 nm provided by the Leonardo^®^ diode laser enable tissue vaporization and blood vessel coagulation, obstructing the blood supply to the fibroid and facilitating its reduction even if complete myoma vaporization is not achieved.

The possibility of executing hysteroscopic laser myolysis in an outpatient setting without anesthesia enables direct patient communication during the procedure, minimizes blood loss, diminishes the risk of fluid absorption, and avoids potential complications such as uterine perforation. The rapid procedure time, anesthesia absence and prompt return to normal activities render HLA a cost-effective therapeutic alternative to treat fibroid.

Despite these promising outcomes, it is crucial to acknowledge the limitations of this report, encompassing its case series design, limited patients' number and brief follow-up period. Equally important is the necessity of a comprehensive discussion with patients and informed consent due to the lack of histopathological analysis before or after HLA, a limitation shared with other techniques such as HIFU or RFA. For this reason, the treated patients in this report were premenopausal with a lower risk of malignancy [62, 63]. Patients with obesity, a history of radiation therapy, retinoblastoma, or past or current tamoxifen treatment were excluded due to their elevated uterine sarcoma risk [64, 65].

In conclusion, our case series demonstrates that the hysteroscopic laser myolysis using the Leonardo^®^ diode laser DWLS is a safe and effective strategy for managing heavy menstrual bleeding of patients with FIGO type 1 or 2 submucous fibroids in an outpatient setting. The direct visualization allowed by hysteroscopy facilitates precise targeting of fibroids, which is not always possible with other techniques. Moreover, the ability to perform laser myolysis in an outpatient setting without general anesthesia or sedation offers increased patient comfort and reduces potential risks associated with anesthesia. Notwithstanding the encouraging results, additional research employing diverse study designs, larger patient cohorts, and longer follow-up durations is imperative to validate these findings and evaluate the long-term outcomes and potential complications associated with this technique.

## Data Availability

The data that support the findings of this study are available on request from the corresponding author.
